# Uptake of multi-level HIV interventions and HIV-related behaviours among young people in rural South Africa

**DOI:** 10.1371/journal.pgph.0003258

**Published:** 2024-05-31

**Authors:** Nondumiso Mthiyane, Maryam Shahmanesh, Andrew Copas, Natsayi Chimbindi, Jaco Dreyer, Thembelihle Zuma, Nuala McGrath, Kathy Baisley, Sian Floyd, Isolde Birdthistle, Lorraine Sherr, Janet Seeley, Guy Harling

**Affiliations:** 1 Africa Health Research Institute, Durban, KwaZulu-Natal, South Africa; 2 Institute for Global Health, University College London, London, United Kingdom; 3 School of Nursing & Public Health, University of KwaZulu-Natal, Durban, South Africa; 4 School of Primary Care, Population Sciences and Medical Education and Department of Social Statistics and Demography, University of Southampton, Southampton, United Kingdom; 5 Department of Infectious Disease Epidemiology, London School of Hygiene and Tropical Medicine, London, United Kingdom; 6 Department of Global Health and Development, London School of Hygiene and Tropical Medicine, London, United Kingdom; 7 MRC/Wits Rural Public Health & Health Transitions Research Unit (Agincourt), University of the Witwatersrand, Johannesburg, South Africa; 8 Department of Epidemiology & Harvard Center for Population and Development Studies, Harvard T.H. Chan School of Public Health, Boston, MA, United States of America; University of California San Francisco, UNITED STATES

## Abstract

Combination HIV prevention packages have reduced HIV incidence and improved HIV-related outcomes among young people. However, there is limited data on how package components interact to promote HIV-related prevention behaviours. We described the uptake of HIV prevention interventions supported by Determined, Resilient, Empowered, AIDS-free, Motivated and Safe (DREAMS) Partnership and assessed the association between uptake and HIV-related behaviours among young people in rural KwaZulu-Natal, South Africa. We analysed two cohorts followed from May 2017 to December 2019 to evaluate the impact of DREAMS, covering 13–29 year-old females, and 13–35 year-old males. DREAMS interventions were categorised as healthcare-based or social. We described the uptake of interventions and ran logistic regression models to investigate the association between intervention uptake and subsequent protective HIV-related outcomes including no condomless sex and voluntary medical male circumcision (VMMC). For each outcome, we adjusted for socio-demographics and sexual/pregnancy history and reported adjusted odds ratios (aOR) and 95% confidence intervals (CI). Among 5248 participants, uptake of healthcare interventions increased from 2018 to 2019 by 8.1% and 3.7% for males and females respectively; about half of participants reported receiving both healthcare and social interventions each year. The most utilised combinations of interventions included HIV testing and counselling, school-based HIV education and cash transfers. Participation in social interventions only compared to no intervention was associated with reduced condomless sex (aOR = 1.60, 95%CI: 1.03–2.47), while participation in healthcare interventions only was associated with increased condomless sex. The uptake of interventions did not significantly affect subsequent VMMC overall. Among adolescent boys, exposure to school-based HIV education, cash transfers and HIV testing and counselling was associated with increase in VMMC (aOR = 1.79, 95%CI: 1.04–3.07). Multi-level HIV prevention interventions were associated with an increase in protective HIV-related behaviours emphasizing the importance of accessible programs within both school and community settings for young people.

## Background

Young people in South Africa continue to be infected with HIV, with adolescent girls and young women (AGYW) being at highest risk of HIV compared to males [1]. More than a quarter of new HIV infections occur among AGYW aged 15–24 years compared to 10% occurring among adolescent boys and young men (ABYM) in the same age group [1], and a range of factors including individual, interpersonal and community-level contribute to the high risk of HIV among young people. Despite ABYM being at lower risk of HIV than AGYW, they are still at risk due to several risky sexual behaviours including having multiple sex partners, condomless sex, and not getting treated for sexually transmitted infections (STI) including HIV [2]. This highlights a substantial HIV risk in both AGYW and ABYM, and as a result, there have been increasing calls for combination interventions that address the risk at multiple levels of influence to prevent HIV in young people [3, 4].

Previous studies have shown how combination approaches are effective in improving HIV-related outcomes [5, 6]. The DREAMS (Determined, Resilient, Empowered, AIDS-free, Mentored and Safe) Partnership is one of the combination strategies for HIV prevention in sub-Saharan Africa, aimed at reducing new HIV infections and HIV-related vulnerabilities among AGYW by addressing underlying multi-level factors [7]. DREAMS packages include biomedical, behavioural and contextual interventions that are delivered directly to AGYW, their families and communities to reduce HIV risk in AGYW and in their male sex partners through strengthening HIV services for men.

While DREAMS has been well received in different settings, the uptake of interventions may vary by type of intervention (i.e., interventions that address behavioural or social factors) and the manner in which interventions are delivered may have resulted in young people receiving different combinations of interventions [8, 9]. Prior work has shown high uptake of multiple interventions among AGYW who were invited to participate in DREAMS [10]. However, there is limited evidence regarding the uptake of specific combinations of interventions and how different components of combined interventions influence the behaviour that could potentially reduce HIV risk in young people.

In South Africa, low uptake of behavioural and biomedical interventions including HIV testing, voluntary medical male circumcision (VMMC) and condom use was reported among youth before the roll-out of DREAMS [11, 12]. These interventions are fundamental to combination HIV prevention in high prevalence settings [13], and therefore, it is crucial to understand how different components of the combination intervention interact to promote safe HIV-related behaviours. This analysis aimed to describe the uptake of HIV interventions delivered in rural South Africa as a multi-sectoral approach to reducing new infections in AGYW; and investigate the association between uptake of multi-level interventions and HIV-related behaviours. Specifically, we looked at the association between multi-level intervention and subsequent use of essential biomedical and behavioural interventions including condom use and VMMC. Consistent condom use and VMMC play a crucial role in reducing HIV risk during sexual intercourse.

## Methods

### Setting

This study was conducted in the southern area of Africa Health Research Institute (AHRI) Population Intervention Platform (PIP) located in uMkhanyakude district, KwaZulu-Natal. Established in 2000, the AHRI PIP covers an area of 438 km^2^, with a population of approximately 100,000 people who are members of 12,000 households [14]. The area is largely rural with one town with an approximate population of 30,000 people. AHRI conducts annual household-based surveys to collect information on births, deaths, and migration patterns from all household members, including non-residents. In addition, residents aged ≥15 years are invited to participate in an annual HIV serosurvey, and to complete a questionnaire on general health and sexual behaviour.

### Data source

**Fig 1:**
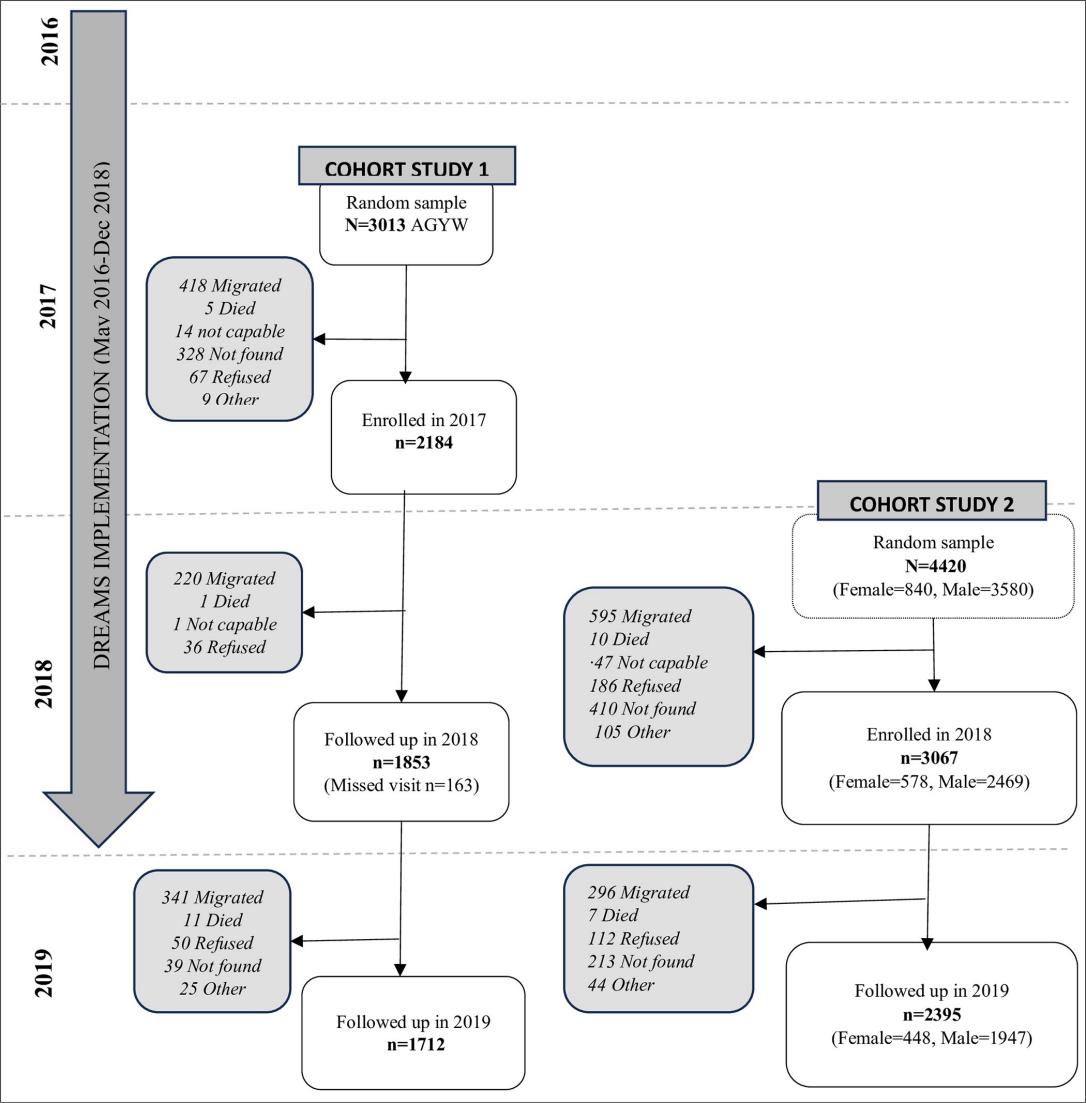
Recruitment and participation flowchart.

Data were drawn from two cohort studies which evaluated the impact of DREAMS among young people. DREAMS was implemented in the uMkhanyakude district of KwaZulu-Natal from May 2016 until December 2018; the DREAMS impact evaluation began in May 2017 as indicated in Fig 1. The first cohort study recruited adolescent girls and young women (AGYW) aged 13–22 years who were enrolled in 2017 and followed-up for two years (2018–2019) [15]. The second cohort study recruited adolescent boys and young men (ABYM) aged 13–35 years and young women aged 24–29 years in 2018 and re-interviewed them in 2019 [16]. Both studies used random sampling, stratified by age, sex and area based on a sampling frame of potential participants comprising all residents within the study area; there was no overlap in study participants. Retention rates at follow-up time points were high (above 75%) in both studies.

### Data collection and management

Data were collected by a team of trained fieldworkers using face-to-face and self-administered interviews (i.e., each participant responded to both face-to-face and self-interviews) and captured electronically in REDCap software [17]. In each survey year, a structured questionnaire was used to collect data on general health, uptake of interventions, behaviour, and sexual relationships; study tools were intentionally comparable between studies and over time. Questions about sexual relationships were self-administered to provide privacy, with fieldworker support available if needed. Datasets from the cohort studies were joined together and then merged with other AHRI household datasets containing migration history and household assets information, based on AHRI unique household identifiers and survey year.

To prepare the data for multivariable analysis, we excluded the 2017 survey because it was only available for the first cohort study (AGYW aged 13–22). We then transformed the data for each person from observation points into observation period (2018–2019). The exposure (uptake of healthcare or social interventions) and potential confounders were measured at the start-of-period interview (in 2018), while all outcomes were measured at the end-of-period interview (in 2019).

### Definitions

#### Uptake of interventions

DREAMS supported 16 intervention types in uMkhanyakude, with varying eligibility criteria (Table 1). Several interventions (including HIV testing, condom provision and VMMC) aimed at decreasing the risk of HIV transmission from male sexual partners to AGYW were strengthened through collaboration with different organisations [18]. For example, DREAMS implementing partners worked with the Department of Health to expand HIV testing and treatment for men through twilight testing in late hours, workplace testing and outreach. In this analysis, similar interventions were combined then grouped according to whether they were healthcare or social interventions. Social interventions included community mobilisation, family-focused and economic strengthening interventions. Most social interventions were only provided through DREAMS, but some interventions such as cash transfers and HIV education or Life skills existed before DREAMS. Healthcare interventions included biomedical interventions (e.g., HIV testing, contraception) and those that promote safer sex behaviours (e.g., by making condoms accessible to individuals). Uptake of interventions was defined as self-reported participation in any relevant intervention in the 12 months prior to the start-of-period interview. We then built a composite uptake variable with four levels: never received any intervention; received only social interventions; received only healthcare interventions; and received multi-level (social and healthcare) interventions.

**Table 1:** The description of interventions supported by DREAMS in uMkhanyakude.

Intervention	Description	Eligible population†
**Social**		
Financial literacy training^‡^	Includes saving groups and microfinance program	All
Vocational/business skills training^‡^	Helps young people to get the essential skills needed to start a business.	All
Local program for parenting/caregiving^‡^	Improves parent-child relationships and communication and reduce problem behaviours and emotional distress for both parent/caregiver and child.	All aged 13–19
Cash transfers	Support for school fees, government social grants or unconditional cash transfers to families.	All
Safe spaces^‡^	Places where AGYW meet regularly to talk about their health and other challenges they face in their lives.	AGYW aged 13–24
Mentor program^‡^	Offered in safe spaces where trained mentors meet with groups of girls to discuss economic hardships and health-related challenges.	AGYW aged 13–24
Social assets program^‡^	Helps AGYW build strong relationships with their peer and adults who can offer emotional and material support.	AGYW aged 13–24
HIV education or Life skills	Offered in all schools as part of basic education curriculum. Participants for this intervention included adolescents aged <20 years.	13–19 years
Gender norms and violence prevention related programs^‡^	Include gender-based violence prevention and gender norms-related education and sexual and reproductive health communication and relationship skills. Offered to adolescents in schools and to all age groups in communities	All
**Healthcare**		
HIV testing and counselling services	Include all types of HIV testing (facility-based, mobile and home-based testing).	All
Voluntary medical male circumcision (VMMC)	Removal of foreskin by a trained health professional. The uptake of VMMC was calculated among males previously reported no VMMC.	Males
Condom promotion and provision	Condom promotion/demonstration and provision in health facilities and in communities.	All
Counselling and provision of contraception/family planning	Covers all types of modern contraception including emergency contraception.	All females
Adolescent friendly services	Services designed for adolescents and young adults and delivered in the health facilities.	All aged 13–24
STI screening and treatment	Offered at the health facility.	All
Post-violence care	Designed for victims of violence (sexual, physical, and emotional abuse).	All

†Participants eligible for intervention and included in the analysis

^**‡**^ Interventions which were only provided through DREAMS

#### Outcomes

All outcomes were based on self-reported activity over the 12 months prior to end-of-period interview in 2019. This activity therefore occurred after the start-of-period interview when participants reported uptake of interventions in 2018 (their exposure). The outcomes were no condomless-sex and VMMC incidence.

*No condomless sex w*as defined as not having any acts of condomless sex in the 12 months preceding the end-of-period interview date, i.e., if a participant reported either no sexual activity or having used a condom for all sex acts in the period. Participants were asked if they had any condomless sex either in the past month or past 12 months. Responses from the two questions were combined to create one variable.

*VMMC* was defined as having received any male circumcision following the start-of-period interview, whereby the foreskin was removed. Females and males who reported having previously been circumcised at start-of-period interview were excluded for this outcome.

#### Potential confounding variables

Potential confounders included socio-demographic variables (age, gender, geographic area, household wealth index, education, household food insecurity, migration) and sexual behaviour. Age of participants was grouped into four categories (13–19, 20–24, 25–29, 30–35). Geographic area was classified as either rural or urban (including townships). Household wealth index scores were calculated using principal component analysis based on ownership of household assets and access to safe drinking water and sanitization; scores from the first principal component were divided into tertiles. For education, the highest level of education achieved was categorised as any primary, any secondary and completed secondary/any tertiary. Food insecurity was defined as any report of reducing the size of food portions or skipping meals by any member of a household because there was not enough money to buy food in the past 12 months. Migration was defined as ever having moved home since age of 13. For pregnancy and sexual history, a composite three-level variable was created to reduce multi-collinearity among independent variables as: never had sex; ever had sex but never pregnant; and ever pregnant. All potential confounders were measured in 2018.

### Statistical analysis

We described the baseline characteristics of all participants enrolled in the two cohorts, overall and by type of intervention they received. For each intervention and intervention-level, we calculated uptake for each survey year as the percentage of participants who used the intervention, and we presented the results using bar graphs. The denominator included young people who were eligible for that particular intervention as indicated in Table 1. For example, for parenting program, we included only adolescents aged 13–19. For each participant, we also calculated the count of interventions received within each level. Using UpSet plots [19], we described all possible combinations of interventions and the number of participants who received each combination of interventions.

**Fig 2:**
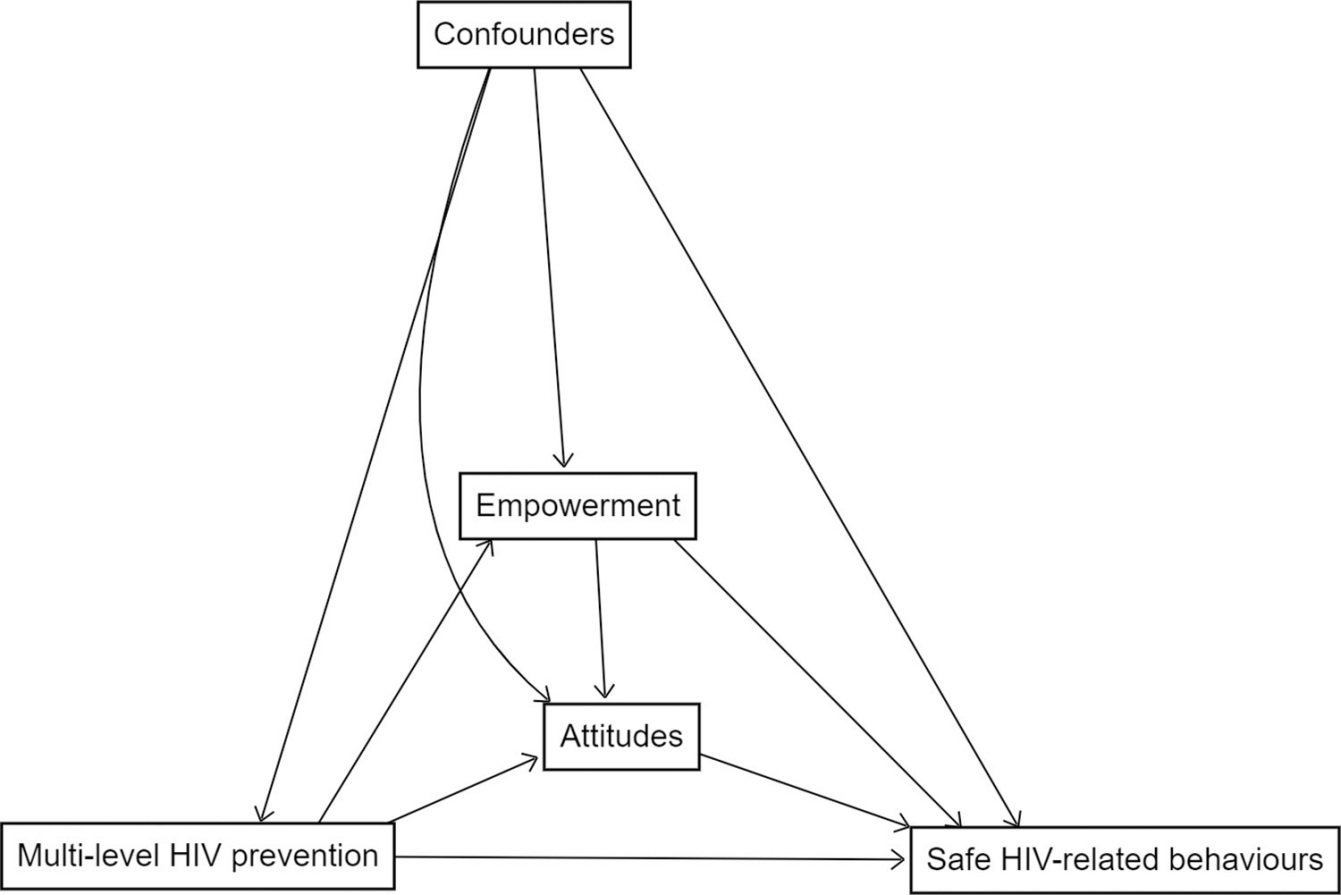
Causal model indicating the hypothesized exposure-outcome relationship.

Guided by our causal model (Fig 2), we examined the association between exposure to multi-level HIV prevention interventions and HIV-related safe behaviours (condom use and VMMC). The model hypothesises that multi-level HIV prevention interventions will increase the uptake of condom use and VMMC through empowerment (i.e., economic empowerment and adequate knowledge of HIV risk) and positive attitude towards the use of HIV prevention services. The model also accounts for potential confounding variables which have been previously shown to be associated with participation in DREAMS interventions [10]. In multivariable analysis for condom use, we adjusted for confounders comprise age, sex, urbanicity, household socio-economic status, food insecurity, level of education, migration history and sexual behaviour. Same covariates (except sex) were also included in the model for VMMC. In the multivariable analyses, we included only participants who had data for 2018–2019 period. For both outcomes, we used ordinary logistic regression model adjusting for potential confounders to examine the association between exposure and outcomes. Since the exposure and confounders were measured in 2018, we assumed that they did not vary between 2018 and 2019. For all multivariable analyses we fitted two models: first including only the exposure variable and second adding all prespecified covariates. Full details are provided in S1 Table. For VMMC analysis, we excluded uptake of VMMC from the exposure to avoid possible complete separation (perfect prediction) of exposure variable by the outcome variable.

We conducted two sub-group analyses to assess possible effect-modification. For all outcomes, we stratified our models by age group (13–19 and 20–35) and participant sex (where possible). We also looked at each intervention and common combinations of interventions as exposure variables to check whether observed associations between multi-level interventions and outcomes were influenced by specific interventions. In this analysis, we first showed the associations between single interventions and outcomes, then showed the associations between combination of interventions and outcomes.

Data cleaning and analysis was done using Stata 14 (College Station, TX, US) and plots were created in R version 3.5.1 [20].

### Ethics approval and consent to participate

The cohort study protocols were approved by the University of KwaZulu-Natal Biomedical Research Ethics Committee (BFC339/19), the London School of Hygiene & Tropical Medicine Research Ethics Committee (REF11835) and the AHRI Somkhele Community Advisory Board. Potential participants were visited in their homes and invited to participate in the study. Participants aged 18 years or older provided a written consent. Parental consent with participant written assent was sought for participants younger than 18 years.

## Results

### Characteristics of participants

A total of 5248 participants were included in the analysis most of whom were aged between 13 and 19 years (Table 2). About 66% of participants were residing in rural areas and 66% had at least secondary education. About a quarter of participants were from households with a low wealth index and had a history of food insecurity. About 20% had ever migrated outside the surveillance area since the age of 13 years. More than half had ever had sex, with 39% of females reporting ever being pregnant. The characteristics of participants differed by intervention exposure. Majority of participants who used social or multi-level interventions were younger than 20, never migrated, or ever had sex, while those who used healthcare interventions only were older (20 and above) and 87% reported ever having had sex.

**Table 2:** Characteristics of participants at baseline, by exposure to intervention.

	All		No intervention		Social		Healthcare		Multi-level	
	N	%	N	%	N	%	N	%	N	%
**Sex**										
Male	2487	47.4	284	52.6	366	39.3	747	46.0	1090	50.7
Female	2761	52.6	256	47.4	566	60.7	877	54.0	1062	49.3
**Age group**										
13–19	2911	55.5	227	42.0	883	94.7	237	14.6	1564	72.7
20–24	1105	21.1	143	26.5	39	4.2	570	35.1	353	16.4
25–29	851	16.2	103	19.1	7	0.8	558	34.4	183	8.5
30–35	381	7.3	67	12.4	3	0.3	259	15.9	52	2.4
**Urbanicity**										
Rural	3438	65.5	345	63.9	638	68.5	1002	61.7	1453	67.5
Peri-urban	1808	34.5	195	36.1	293	31.4	621	38.2	699	32.5
**Household wealth index**										
Low	1293	24.6	131	24.3	240	25.8	391	24.1	531	24.7
Middle	1716	32.7	164	30.4	290	31.1	518	31.9	744	34.6
High	1706	32.5	190	35.2	313	33.6	530	32.6	673	31.3
Unknown	533	10.2	55	10.2	89	9.5	185	11.4	204	9.5
**Migration**										
Never	3744	71.3	352	65.2	860	92.3	792	48.8	1740	80.9
Within PIPSA	475	9.1	43	8.0	46	4.9	201	12.4	185	8.6
External migration	1029	19.6	145	26.9	26	2.8	631	38.9	227	10.5
**Highest educational attainment (four categories)**										
None or Some primary	418	8	53	9.9	137	14.7	81	5.0	147	6.8
Some secondary	3490	66.7	295	55.1	771	82.7	694	42.9	1730	80.5
Completed secondary	1324	25.3	187	35.0	24	2.6	841	52.0	272	12.7
**Skipped meals in past 12m**										
No	3874	73.8	406	75.5	754	80.9	1154	71.1	1560	72.5
Yes	1364	26	131	24.3	176	18.9	468	28.8	589	27.4
**Ever had sex, ever pregnant composite variable**										
Never had sex	2324	44.3	234	43.3	812	87.1	144	8.9	1134	52.7
Ever sex, never pregnant	1696	32.3	219	40.6	100	10.7	767	47.2	610	28.3
Ever pregnant	1098	20.9	72	13.3	13	1.4	657	40.5	356	16.5
Unknown	130	2.5	15	2.8	7	0.8	56	3.4	52	2.4

### Uptake of interventions

**Fig 3:**
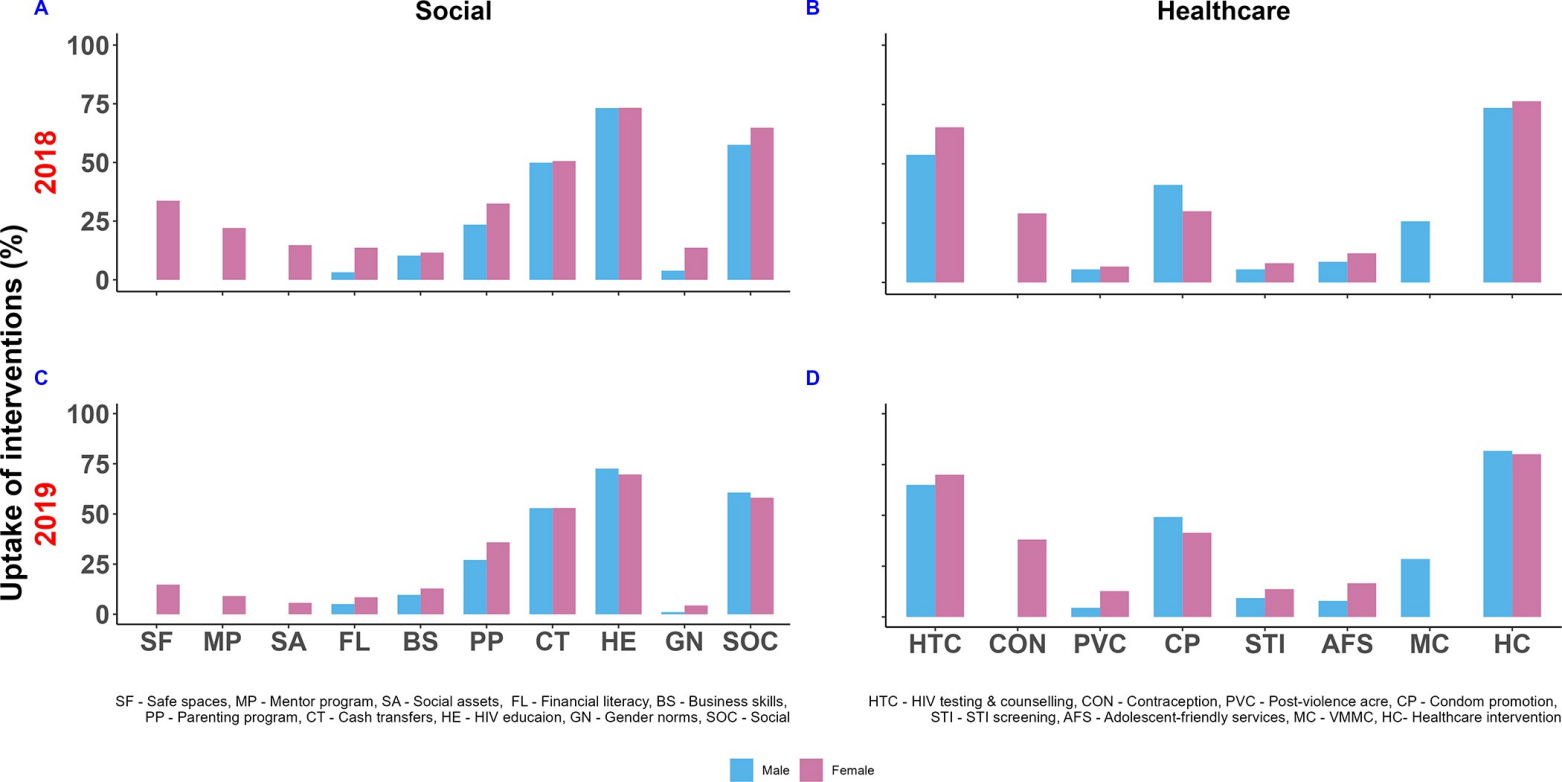
Uptake of interventions, by age, gender and year of survey.

Overall, uptake of each of the healthcare and social interventions was high across gender, for males and females (Fig 3). Full details are provided in the S2 Table. Among social interventions school-based HIV education had the highest uptake (above 70% in 2018 and 2019), followed by cash transfers with 50% uptake. In 2019, there was a decrease in the uptake of social interventions among females. Among healthcare interventions, HIV testing and counselling had highest uptake (above 60% in 2018 and 2019) followed by condom promotion (36% in 2018 and 45% in 2019).

**Fig 4:**
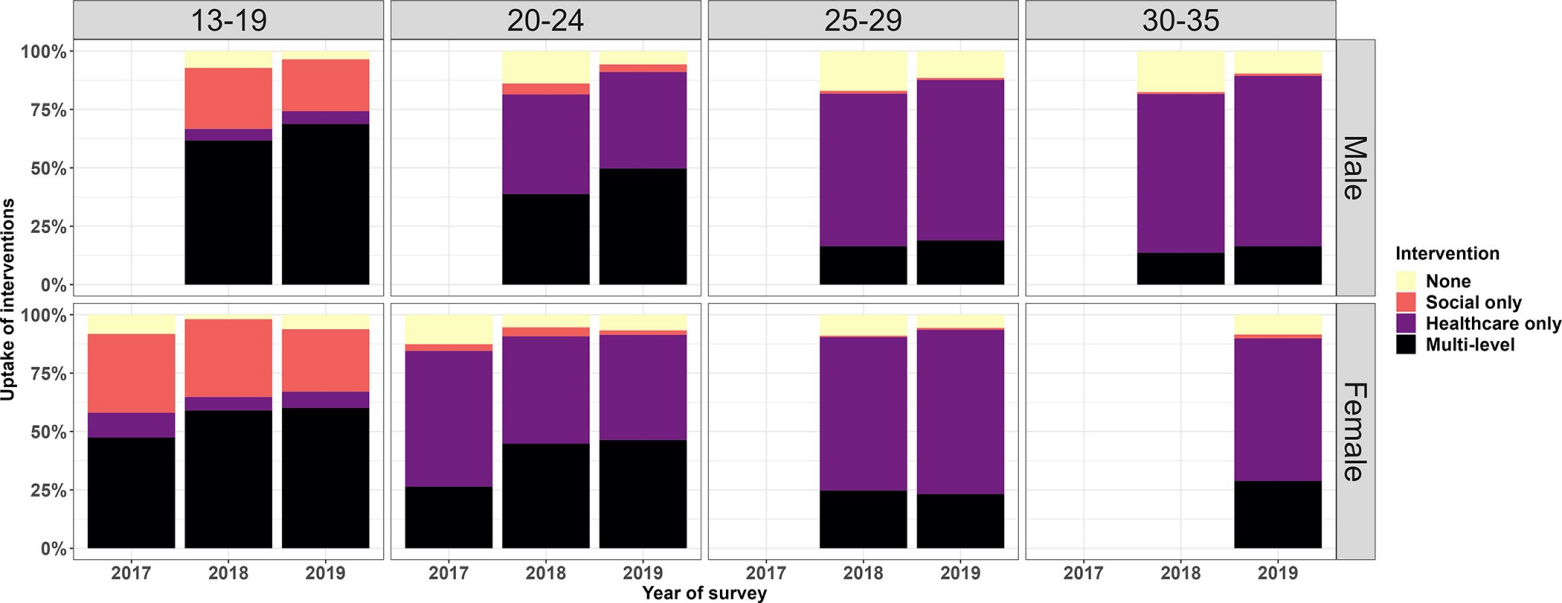
Uptake of multi-level interventions, by age and year of survey.

**Fig 5:**
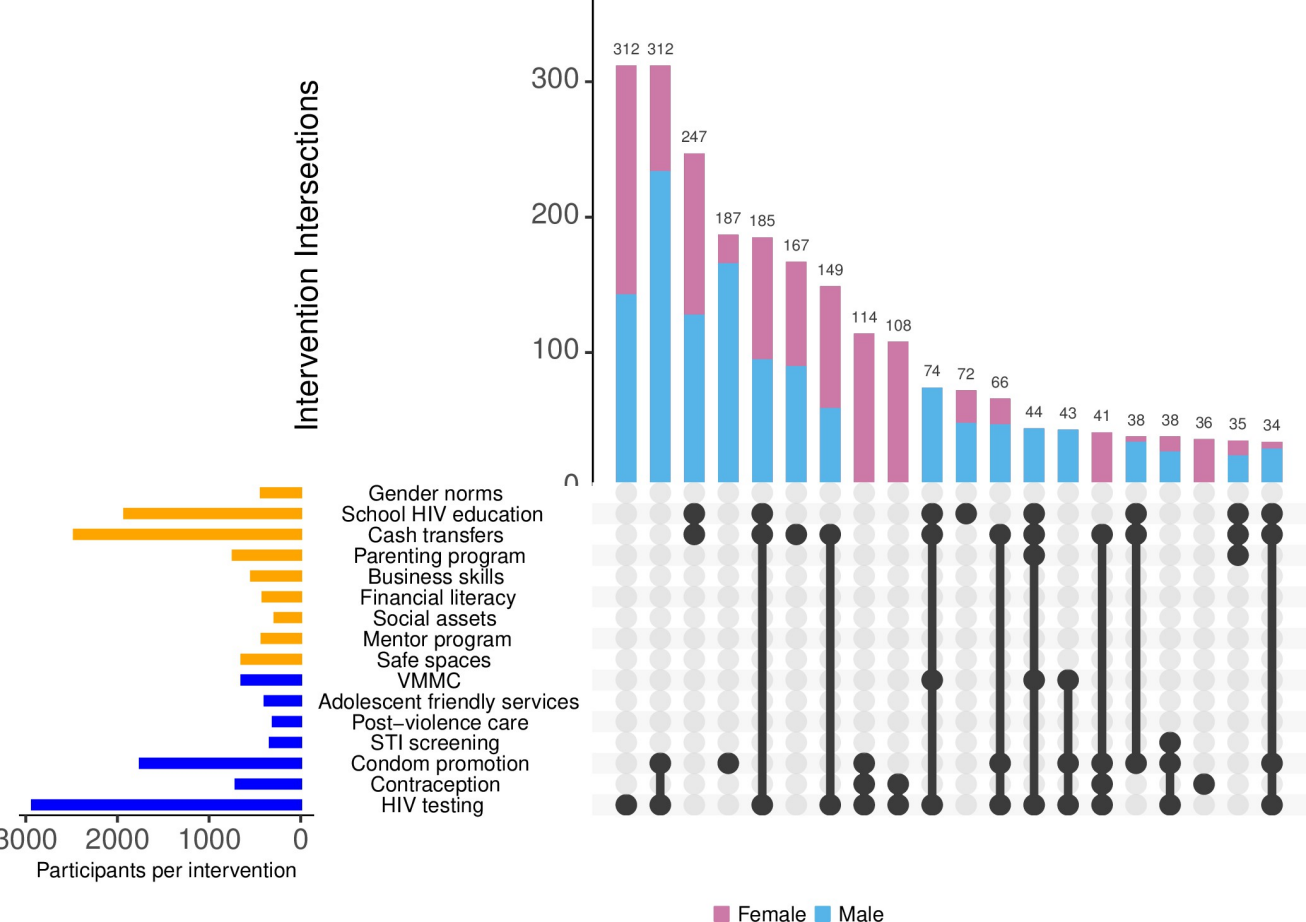
Uptake of combination of HIV prevention interventions in 2018.

Intervention uptake varied by age: more than half of adolescents aged 13–19 years received both healthcare and social interventions in 2018 and 2019, And most adolescents who did not receive both levels of intervention participated in social interventions (Fig 4). Most 20–24 year-olds received either multi-level or only healthcare interventions, whereas most (>80%) of those aged 25 and above received only healthcare interventions. The most common combinations of multi-level interventions were school-based HIV education, cash transfers and HIV testing and counselling, followed by cash transfers and HIV testing (Fig 5). These combinations were common for both males and females (S1 and S2 Figs).

### Association between social and individual-level interventions and subsequent HIV-related behaviours

Table 3 shows the adjusted odd ratios from the regression models that examined the association between uptake of intervention and subsequent behaviour and use of HIV prevention services. Full details are provided in the S4–S6 Tables.

**Table 3:** Association between uptake of healthcare/social interventions and subsequent HIV-related behaviours.

	No condomless-sex (N = 3728) †			VMMC (N = 831) ^‡^		
	n (%)	OR (95% CI)	aOR (95% CI)	n (%)	OR (95% CI)	aOR (95% CI)
**Multi-level Intervention**						
No intervention	139 (51.7)	1	1	24 (21.1)	1	1
Social only	626 (89.7)	8.13 (5.78–11.44)	1.60 (1.03–2.47)	27 (16.8)	0.76 (0.41–1.39)	0.65 (0.32–1.32)
Healthcare only	337 (35.0)	0.50 (0.38–0.66)	0.96 (0.69–1.35)	44 (16.9)	0.76 (0.44–1.33)	1.08 (0.57–2.02)
Multi-level	1230 (68.4)	2.02 (1.56–2.62)	1.14 (0.81–1.60)	76 (25.7)	1.30 (0.77–2.18)	1.21 (0.67–2.19)
**Common interventions and combinations**						
**HIV testing**						
No	1657 (74.9)	1	1	114 (21.6)	1	1
Yes	675 (44.6)	0.27 (0.23–0.32)	0.67 (0.55–0.81)	57 (18.8)	0.84 (0.59–1.20)	1.12 (0.71–1.74)
C**ondom promotion**						
No	1944 (71.5)	1	1	115 (20.7)	1	1
Yes	388 (38.5)	0.25 (0.21–0.29)	0.69 (0.57–0.83)	56 (20.4)	0.99 (0.21–0.32)	1.38 (0.88–2.19)
**Contraception**						
No	220 (41.8)					
Yes	1020 (74.1)	0.25 (0,20–0.31)	0.72 (0.56–0.94)			
**School-based HIV education** ^**⁋**^						
No	561 (74.9)	1	1	33 (24.3)	1	1
Yes	1223 (86.3)	2.11 (1.69–2.64)	1.82 (1.42–2.32)	59 (22.9)	0.93 (0.57–1.51)	0.89 (0.53–1.49)
**Cash transfers**						
No	938 (50.2)	1	1	89 (19.3)	1	1
Yes	1394 (75.0)	2.99 (2.60–3.43)	1.41 (1.19–1.67)	82 (22.2)	1.20 (0.85–1.68)	0.97 (0.64–1.47)
**School-based HIV education & cash transfers** ^**⁋**^						
No	816 (78.6)	1	1	41 (23.2)	1	1
Yes	968 (85.8)	1.65 (1.32–2.06)	1.42 (1.12–1.80)	51 (23.5)	1.02 (0.64–1.63)	1.04 (0.62–1.72)
**School-based HIV education & cash transfers & HIV testing** ^**⁋**^						
No	1254 (83.4)	1	1	63 (20.8)	1	1
Yes	530 (80.1)	0.80 (0.63–1.01)	0.72 (0.57–0.93)	29 (31.9)	1.78 (1.06–3.00)	1.79 (1.04–3.07)
**School-based HIV education & HIV testing** ^**⁋**^						
No	1101 (84.2)	1	1	57 (19.9)	1	1
Yes	683 (79.6)	0.73 (0.59–0.92)	0.64 (0.50–0.81)	35 (32.4)	1.93 (1.17–3.16)	1.98 (1.18–3.33)
**Cash transfers & HIV** testing						
No	1583 (60.9)	1	1	124 (18.8)	1	1
Yes	749 (66.4)	0.63 (0.50–0.78)	0.80 (0.67–0.96)	47 (27.2)	1.61 (1.09–2.37)	1.45 (0.96–2.18)
**HIV testing & Condom promotion**						
No	2058 (68.9)	1	1	137 (21.0)	1	1
Yes	274 (36.9)	0.26 (0.22–0.31)	0.67 (0.55–0.81)	34 (18.9)	0.87 (0.58–1.33)	1.12 (0.68–1.83)
**Cash transfers & condom promotion**						
No	2073 (64.0)	1	1	141 (19.9)	1	1
Yes	259 (52.8)	0.63 (0.52–0.76)	0.59 (0.47–0.74)	30 (24.8)	1.33 (0.85–2.09)	1.12 (0.68–1.83)

**†**Adjusted for age, sex, location, SES, food insecurity, education, migration and sexual and pregnancy history.

^**‡**^ Adjusted for age, location, SES, food insecurity, education, migration and sexual history

^⁋^The denominator (calculated among participants below <20) is 2166 for condomless sex and 394 for VMMC.

#### No condomless sex

In the multivariable analyses, 379 (9.2%) participants were excluded due to missing data (non-response or missed 2018 survey). Overall, participating in social interventions alone, relative to no intervention, was associated with a higher proportion reporting no condomless sex (aOR = 1.60, 95% CI: 1.03–2.47). There was no significant association between participating in healthcare interventions alone or in multi-level interventions and no condomless sex, either overall or by age group or sex. However, when we looked at the exposure to specific interventions or combinations, we found that participating in cash transfers and school-based HIV education was significantly associated with higher odds of no condomless sex (aOR = 1.42, 95% CI: 1.12–1.80), while exposure to both HIV testing and counselling and condom promotion (combined) was significantly associated with lower odds of no condomless sex (aOR = 0.67, 95% CI: 0.55–0.81). Similar results were observed even when these interventions were separated. Among females, participating in contraception was associated with lower odds of no condomless sex (aOR = 0.72, 95% CI: 0.56–0.94) compared to not participating in contraception.

#### VMMC

Overall, there was little evidence that uptake of healthcare and social interventions was associated with an increase in VMMC in the overall sample, after adjusting for potential confounders (Table 3). There was no obvious effect modification by age group. When looking at the specific combinations of interventions (Table 3), we found that exposure to school-based HIV education, cash transfers and HIV testing and counselling (aOR = 1.79, 95% CI:1.04–3.07) or school-based HIV education and HIV testing and counselling (aOR = 1.98, 95% CI:1.18–3.33) was associated with increase in VMMC among adolescent boys. School-based HIV education and cash transfers (without HIV testing and counselling) or HIV testing and counselling alone were not associated with VMMC.

## Discussion

In this longitudinal study, we found an increase over time in the uptake of individual level interventions, with HIV testing and counselling having the highest uptake in both males and females. School-based HIV education and cash transfers were the most used social interventions. Most adolescents participated in both healthcare and social interventions while the majority of young people aged ≥20 years participated only in healthcare interventions. We also found that common combinations of multi-level interventions that were used by young people included school-based HIV education, cash transfers and HIV testing and counselling. The uptake of these multi-level interventions was associated with subsequently higher VMMC in younger boys only, while uptake of social interventions was associated with reduced condomless sex in young people.

The high levels of uptake of both healthcare reported in this study in the younger group are consistent with a previous study that measured uptake of DREAMS interventions among AGYW in this setting [10]. The high uptake of social interventions among adolescents may be driven by the delivery approach that used schools to recruit young people who needed services and deliver interventions such as violence prevention education [9]. In this analysis we also identified HIV testing and counselling, school-based HIV education and cash transfers as the common combination of multi-level interventions that young people received. The increase in HIV testing in this setting may have been partly influenced by home-based (including mobile) HIV testing which is easier to access than facility-based services [21]. While these three interventions can be delivered simultaneously to young people, being exposed to cash transfers and comprehensive HIV education has been shown to be associated with increased knowledge of HIV and a positive attitude towards HIV prevention in adolescents [22, 23]. The frequency of in-school interventions in this sample highlights that adolescents in school have better access to multiple HIV prevention interventions compared out-of-school peers [24]. This suggests both that schools can play an important part in increasing accessibility and use of HIV prevention services, and that provision of services to out-of-school youth will require greater effort.

Despite the low levels of VMMC uptake observed in this study, adolescent boys who received multi-level interventions were more likely to participate in VMMC than those who did not receive any intervention. The referrals to VMMC services following a home-based HIV testing and school-based VMMC campaigns run by the Department of Health in the study area when DREAMS was implemented may have contributed to the increase in VMMC. The evidence from a study conducted in South Africa and Uganda showed an increase in male circumcision following home-based HIV testing among HIV-negative men who received text message and lay counsellors support [25]. These findings highlight the important role that multi-level interventions can play in improving health seeking behaviours among young people who have limited access to healthcare and those harder to reach (e.g., young men) due to barriers to accessing care [26, 27].

The uptake of social interventions only (particularly cash transfers and school-based HIV education), and combinations of social and healthcare interventions prior to adjustment for confounding, was associated with significantly less subsequent condomless sex. Studies have shown that exposure to interventions such as cash transfers and school-based interventions can cause young people to delay their sexual debut [28, 29]. On the contrary, exposure to healthcare interventions such as HIV testing and counselling, condom promotion and contraception was associated with increase in subsequent condomless sex. This finding is consistent with a study that found low consistent condom use following HIV testing among young people in Kenya [30]. In our study, young people who received only healthcare interventions may have been exposed to HIV testing and counselling through other healthcare interventions such as antenatal care, family planning and VMMC. Their perceptions of HIV risk following exposure to interventions that are linked to low risk can influence the decision to not use a condom (e.g., VMMC) [31, 32]. This finding also suggests that wide-scale promotion and distribution of condoms observed in this setting may not be sufficient and needs to incorporate strategies to clarify social misconceptions about condom use. Furthermore, the value of a dual contraceptive method in HIV prevention and transmission needs to be emphasized to young women accessing family planning services.

Although in this study we did not classify whether condomless sex was with a regular or casual partner, the decision not to use condoms could also be influenced by the type of sexual relationship a person is involved in [33–36]. Previous studies conducted in this rural setting found that low condom use at last sex was associated with having a regular sexual partner or an older partner [37, 38]. Thus, programs that promote condom use should also consider the influence of sexual relationships dynamics on consistent condom use.

Our study has some limitations. First, the data on uptake of interventions and outcomes were self-reported and this could have introduced misclassification bias. Second, the uptake of post-violence care services may have been underestimated as it was calculated among all participants. Although data on violence experiences was available, it was difficult to identify individuals who might have needed post-violence care services. Third, the observed associations between uptake of interventions and outcomes may be influenced by intervention fidelity which we could not measure in our study. Fourth, loss to follow-up was significantly high among participants aged 20–24, who completed secondary school, ever out-migrated and sexually active (S7 Table). Differential loss to follow-up may have introduced selection bias and overestimation of outcomes. However, our sample size and retention rate were high, likely reducing the degree of bias in the study findings. Lastly, although there is temporal ordering of exposure and outcome in our analysis, there may be residual confounding, and thus caution should be taken in interpreting the associations observed in this study as causal effects.

The study also has some strengths. This is the first study in this setting to identify the specific combinations of interventions that are commonly received by male and female youth and to investigate whether different components (social vs healthcare) of combination intervention affect HIV-related behaviours. We also identified interventions that may work well together to improve HIV-related behaviours in adolescents and young adults. However, further research is necessary to test our hypothesised causal mechanism, that is, the influence of empowerment and attitudes on the relationship between multi-level HIV prevention interventions and safe HIV-related behaviours. Furthermore, although most interventions were intended for AGYW, the data from two cohort studies allowed us to compare uptake of interventions between adolescents and young adults.

## Conclusions

There is evidence that adolescents who receive school-based HIV education and child support and other cash transfers also participate in HIV testing and counselling which supports the use of schools as an ideal platform to deliver HIV prevention interventions to adolescents. Participating in multi-level interventions is associated with positive change in HIV-related behaviours that are fundamental to HIV prevention and transmission. Additionally, these findings emphasize the need for tailored HIV prevention strategies for older youth not in school to enhance their uptake of multi-level HIV interventions. Peer-led and digital interventions could help in promoting and expanding access to social-level interventions for young people who are not in school. Further research is needed to understand the association of multi-level HIV interventions and condom use in the context of sexual relationship dynamics.

## Supporting information

S1 Fig Uptake of combination of HIV prevention interventions in 2017, among AGYW. (PDF)

S2 Fig Uptake of combination of HIV prevention interventions in 2019. (PDF)

S1 Table Logistic models adjusting for exposure and potential confounders. (DOCX)

S2 Table Uptake of interventions (percentage of participants who used the intervention), by sex and year of survey. (DOCX)

S3 Table Association between uptake of social/healthcare interventions and no condomless-sex. (DOCX)

S4 Table Association between uptake of social/healthcare interventions and no condomless-sex, by age and sex. (DOCX)

S5 Table Association between uptake of individual/community-level interventions and VMMC, by age. (DOCX)

S6 Table Association between uptake of interventions and HIV-related behaviours among adolescents and young adults. (DOCX)

S7 Table Factors associated with lost to follow-up among adolescents and young adults enrolled in the cohort studies. (DOCX)

## Abbreviations

DREAMS: Determined Resilient Empowered AIDS-free and Safe
ART: antiretroviral therapy
VMMC: voluntary medical male circumcision
AGYW: adolescent girls and young women
AHRI: Africa Health Research Institute
