# Supplementary figures and images for: Uptake of multi-level HIV interventions and HIV-related behaviours among young people in rural South Africa
Source: PLOS Glob Public Health. 2024 May 31;4(5):e0003258. doi: 10.1371/journal.pgph.0003258 (PMC11142690; doi:10.1371/journal.pgph.0003258)

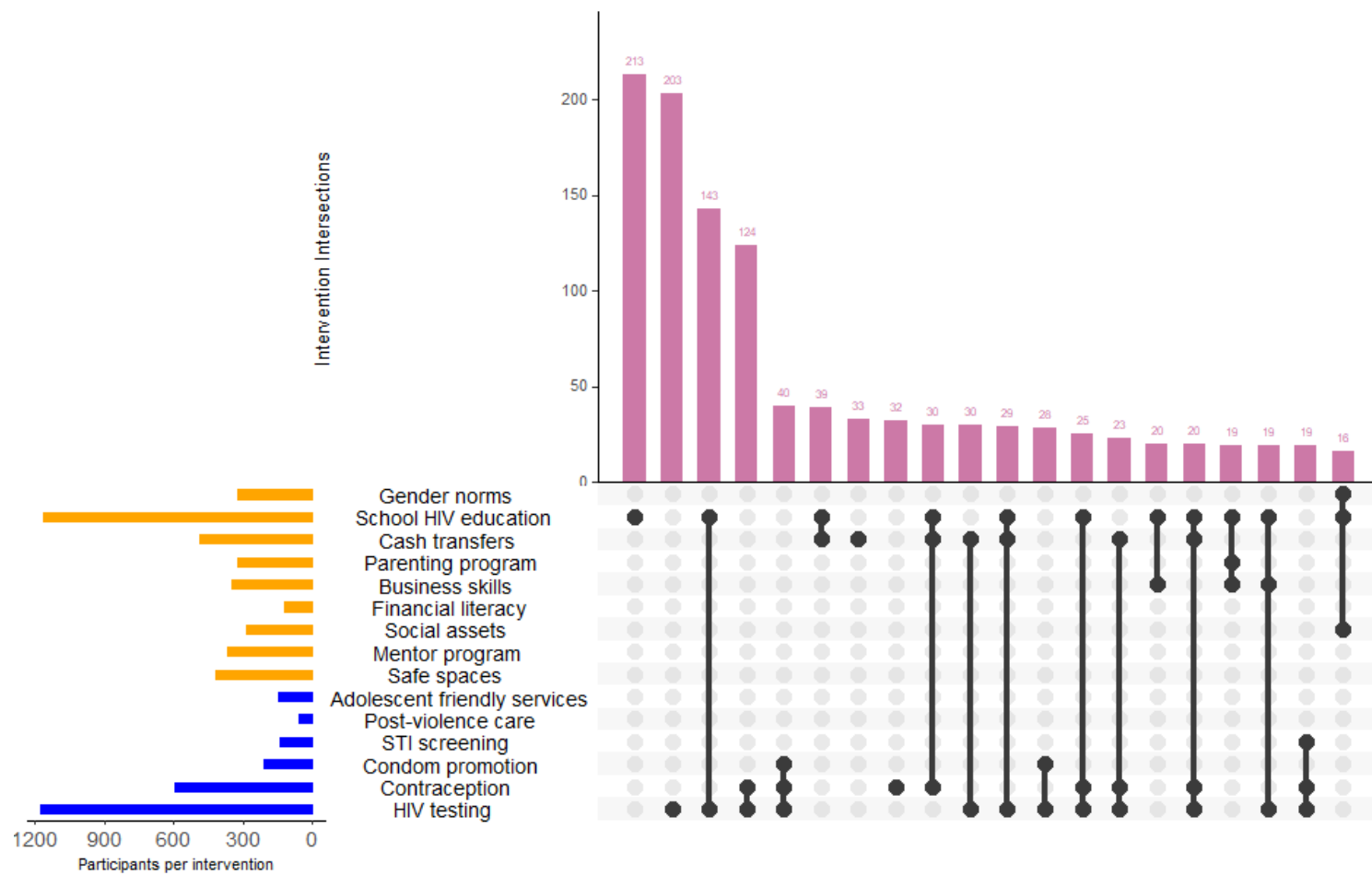

Supplement: S1 Fig — (PDF) [file pgph.0003258.s001.pdf]

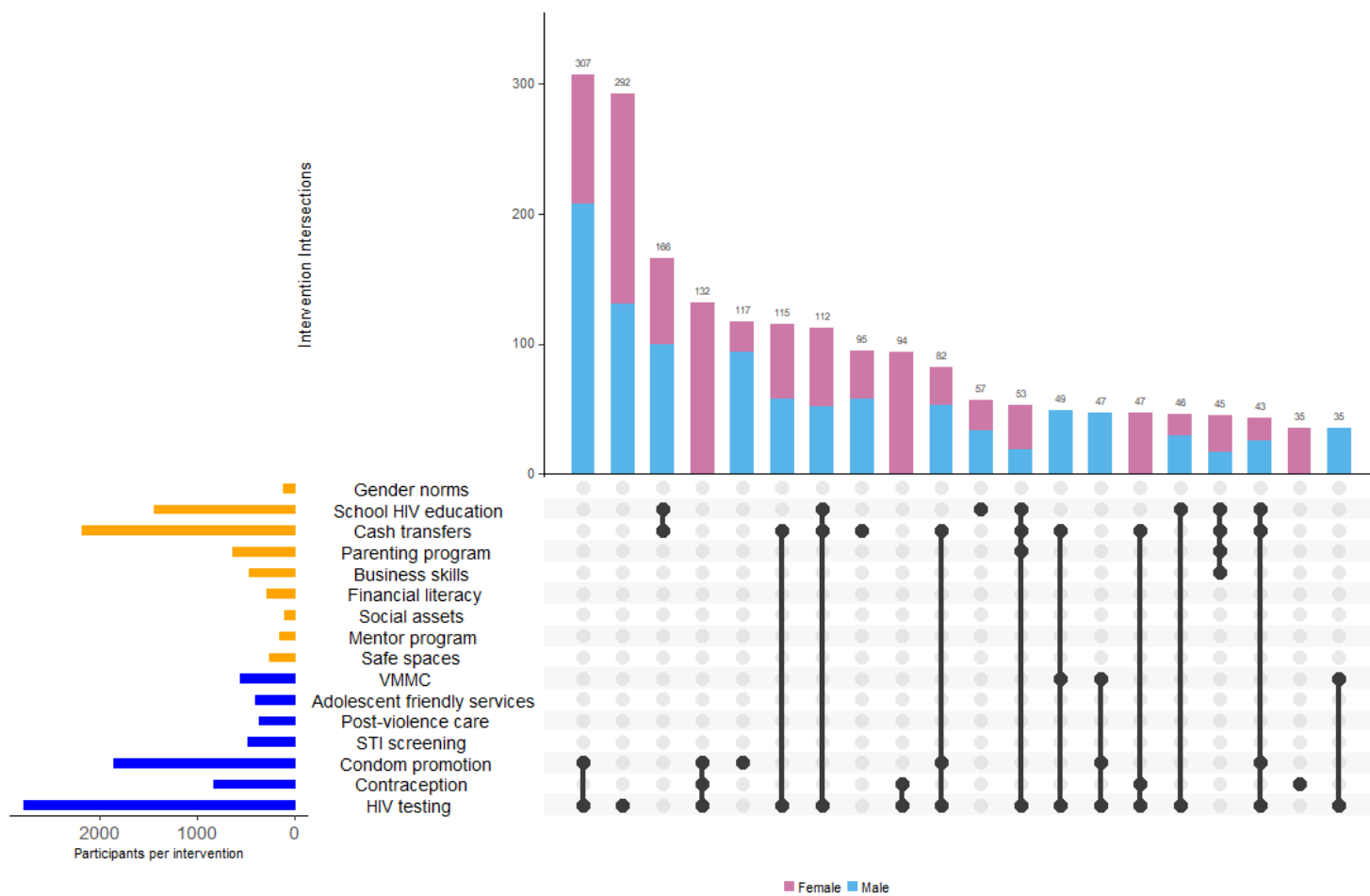

Supplement: S2 Fig — (PDF) [file pgph.0003258.s002.pdf]
