# Supplementary material for: Uptake of multi-level HIV interventions and HIV-related behaviours among young people in rural South Africa
Source: PLOS Glob Public Health. 2024 May 31;4(5):e0003258. doi: 10.1371/journal.pgph.0003258 (PMC11142690; doi:10.1371/journal.pgph.0003258)
Supplement: S1 Table — (DOCX) [file pgph.0003258.s003.docx]

**S1 Table. Logistic models adjusting for exposure and potential confounders**

|  | **2019** | |  |
| --- | --- | --- | --- |
| **2018** | **No condomless sex** | **VMMC** | **Notes** |
| **Exposure** |  |  |  |
| **Social interventions** |  |  | Relevant interventions were collapsed into one variable with 4 categories (No intervention, social only, individual-level only & multi-level) |
| Safe spaces | **✓** |  |  |
| Mentor program | **✓** |  |  |
| Social assets | **✓** |  |  |
| Business skills training | **✓** | **✓** |  |
| Financial literacy | **✓** | **✓** |  |
| Cash transfers | **✓** | **✓** |  |
| Parenting program | **✓** | **✓** |  |
| Violence prevention | **✓** | **✓** |  |
| School-based HIV education | **✓** | **✓** |  |
| **Healthcare interventions** |  |  |  |
| HIV testing & counselling | **✓** | **✓** |  |
| Condom promotion | **✓** | **✓** |  |
| Contraception | **✓** |  |  |
| Post-violence care | **✓** | **✓** |  |
| STI screening & treatment | **✓** | **✓** |  |
| VMMC | **✓** |  |  |
| Adolescent & youth friendly services | **✓** | **✓** |  |
| **Confounders** |  |  |  |
| Age | **✓** | **✓** | Age was not adjusted for in the sub-group analysis involving only adolescents. |
| Sex | **✓** |  |  |
| Urbanicity | **✓** | **✓** |  |
| Education level | **✓** | **✓** |  |
| Socio-economic status | **✓** | **✓** |  |
| Food insecurity | **✓** | **✓** |  |
| Migration | **✓** | **✓** |  |
| Ever had sex/pregnancy | **✓** | **✓** |  |
