# Supplementary material for: Uptake of multi-level HIV interventions and HIV-related behaviours among young people in rural South Africa
Source: PLOS Glob Public Health. 2024 May 31;4(5):e0003258. doi: 10.1371/journal.pgph.0003258 (PMC11142690; doi:10.1371/journal.pgph.0003258)
Supplement: S2 Table — (DOCX) [file pgph.0003258.s004.docx]

**S2 Table. Uptake of interventions (percentage of participants who used the intervention), by sex and year of survey**

|  | **ALL** | | | **MALES** | | **FEMALES** | |
| --- | --- | --- | --- | --- | --- | --- | --- |
|  | **2017** | **2018** | **2019** | **2018** | **2019** | **2018** | **2019** |
|  | **%** | **%** | **%** | **%** | **%** | **%** | **%** |
|  | **N=2184** | **N=4917** | **N=4107** | **N=2487** | **N=1947** | **N=2430** | **N=2160** |
| HIV testing & counselling | 53.8 | 59.5 | 67.6 | 53.8 | 65 | 65.4 | 70 |
| Counselling and provision of family planning/contraception * | 27.2 | 29.1 | 38.1 | . | . | 29.1 | 38.1 |
| Post-violence care | 2.8 | 6.1 | 8.8 | 5.5 | 4.5 | 6.7 | 12.7 |
| Condom promotion and provision | 9.7 | 35.6 | 45.1 | 41.1 | 49.2 | 30 | 41.4 |
| STI screening and treatment | 6.4 | 6.8 | 11.6 | 5.5 | 9.3 | 8.1 | 13.7 |
| Adolescent friendly services† | 6.6 | 10.6 | 12.7 | 8.7 | 7.9 | 12.3 | 16.6 |
| Voluntary medical male circumcision‡ | . | 25.8 | 28.5 | 25.8 | 28.5 | . | . |
| Uptake of at least one healthcare intervention | 64.8 | 75 | 80.9 | 73.6 | 81.7 | 76.4 | 80.1 |
| Safe spaces § | 19.1 | 33.7 | 14.8 | . | . | 33.7 | 14.8 |
| Mentor program § | 17 | 22.1 | 9.1 | . | . | 22.1 | 9.1 |
| Social assets§ | 13 | 14.8 | 5.7 | . | . | 14.8 | 5.7 |
| Financial literacy training | 5.7 | 8.4 | 6.9 | 3.2 | 5.1 | 13.7 | 8.5 |
| Vocational/business skills training | 16.1 | 10.9 | 11.4 | 10.3 | 9.7 | 11.6 | 12.9 |
| Local program for parenting/caregiving ¶ | 20.1 | 28.1 | 31.6 | 23.5 | 27.1 | 32.5 | 35.9 |
| Cash transfers | 22.3 | 50.3 | 52.9 | 49.9 | 52.9 | 50.6 | 53 |
| School-based HIV education ¶ | 71.7 | 73.2 | 71.1 | 73.2 | 72.6 | 73.3 | 69.7 |
| Gender norms and violence prevention | 14.8 | 8.8 | 2.8 | 3.9 | 1.1 | 13.7 | 4.4 |
| Uptake of at least one social intervention | 66.1 | 61.1 | 59.3 | 57.5 | 60.7 | 64.8 | 58.1 |

*Calculated among females only: N=2184 in 2017, N=2430 in 2018, N=2160 in 2019

†Calculated among participants aged 13-24 years, N=2184 in 2017, N=3685 in 2018, N=3106 in 2019

‡ Calculated among males, N=2487 in 2018, N=1947 in 2019

§ Calculated among female adolescents aged 13-24 years, N=2184 in 2017, N=1908 in 2018, N=1713 in 2019

¶ Calculated among adolescents aged 13-19 years, N=1623 in 2017, N=2624 in 2018, N=2020 in 2019
