# Supplementary material for: Uptake of multi-level HIV interventions and HIV-related behaviours among young people in rural South Africa
Source: PLOS Glob Public Health. 2024 May 31;4(5):e0003258. doi: 10.1371/journal.pgph.0003258 (PMC11142690; doi:10.1371/journal.pgph.0003258)
Supplement: S3 Table — (DOCX) [file pgph.0003258.s005.docx]

**S3 Table. Association between uptake of social/healthcare interventions and no condomless-sex.**

|  | **Unadjusted OR (95% CI)** | **Model 1 Adjusted OR (95% CI)** | **Model 2**  **Adjusted OR (95% CI)** |
| --- | --- | --- | --- |
| **Intervention** |  |  |  |
| No intervention | 1 | 1 | 1 |
| Social only | 8.13 (5.78 -11.44) | 1.60 (1.03 -2.47) | 1.15 (0.45 - 2.92) |
| Healthcare only | 0.50 (0.38 -0.66) | 0.96 (0.69 -1.35) | 1.11 (0.65 - 1.91) |
| Multi-level | 2.02 (1.56 -2.62) | 1.14 (0.81 -1.60) | 1.23 (0.71 - 2.15) |
| **Recent past condom use** |  |  |  |
| No | 1 |  | 1 |
| Yes | 10.91 (9.24 - 12.89) |  | 2.82 (1.47 - 5.42) |
| **Intervention × recent past condom use** |  |  |  |
| No intervention × No condom use |  |  | 1 |
| Social only × used condom |  |  | 1.38 (0.47 - 4.01) |
| Healthcare only × used condom |  |  | 0.72 (0.35 - 1.48) |
| Multi-level × use condom |  |  | 0.85 (0.42 - 1.72) |
| **Age group** |  |  |  |
| 13 - 19 | 1 | 1 | 1 |
| 20 - 24 | 0.15 (0.13 -0.18) | 0.67 (0.52 -0.86) | 0.71 (0.54 - 0.92) |
| 25 - 29 | 0.10 (0.08 -0.12) | 0.50 (0.37 -0.69) | 0.51 (0.36 - 0.71) |
| 30 - 35 | 0.07 (0.05 -0.09) | 0.43 (0.28 -0.65) | 0.50 (0.31 - 0.78) |
| **Sex** |  |  |  |
| Male | 1 | 1 | 1 |
| Female | 1.26 (1.10 -1.44) | 1.34 (1.06 -1.71) | 1.32 (1.02 - 1.69) |
| **Geographic area** |  |  |  |
| Rural | 1 | 1 |  |
| Urban | 0.78 (0.68 -0.89) | 1.10 (0.91 -1.31) | 1.08 (0.39 - 1.31) |
| **Highest educational attainment** |  |  |  |
| None or Some primary | 1 | 1 | 1 |
| Some secondary | 0.57 (0.41 -0.80) | 0.60 (0.39 -0.92) | 0.56 (0.36 - 0.89) |
| Completed secondary | 0.14 (0.10 -0.20) | 0.61 (0.39 -0.96) | 0.57 (0.35 - 0.92) |
| **Migration** |  |  |  |
| Never | 1 | 1 | 1 |
| Within PIPSA | 0.34 (0.27 -0.43) | 0.90 (0.68 -1.20) | 0,87 (0.65 - 1.18) |
| External migration | 0.22 (0.18 -0.26) | 1.14 (0.88 -1.46) | 1.13 (0.86 - 1.46) |
| **Household wealth index** |  |  |  |
| Low | 1 | 1 | 1 |
| Middle | 0.96 (0.80 -1.15) | 0.94 (0.75 -1.19) | 0.92 (0.72 - 1.17) |
| High | 0.94 (0.79 -1.13) | 0.88 (0.70 -1.12) | 0.86 (0.67 - 1.10) |
| Unknown | 0.83 (0.66 -1.04) | 0.82 (0.61 -1.11) | 0.84 (0.61 - 1.40) |
| **Food insecurity** |  |  |  |
| No | 1 | 1 | 1 |
| Yes | 0.66 (0.56 -0.77) | 0.82 (0.67 -1.01) | 0.83 (0.67 - 1.03) |
| **Ever had sex, ever been pregnant** |  |  |  |
| Never | 1 | 1 | 1 |
| Ever had sex, never pregnant | 0.04 (0.03 -0.05) | 0.07 (0.05 -0.09) | 0.11 (0.08 - 0.15) |
| Ever pregnant | 0.05 (0.04 -0.06) | 0.07 (0.05 -0.10) | 0.12 (0.09 - 0.17) |
| Unknown | 0.07 (0.04 -0.13) | 0.12 (0.06 -0.22) | 0.08 (0.01 - 0.45) |
