# Supplementary material for: Uptake of multi-level HIV interventions and HIV-related behaviours among young people in rural South Africa
Source: PLOS Glob Public Health. 2024 May 31;4(5):e0003258. doi: 10.1371/journal.pgph.0003258 (PMC11142690; doi:10.1371/journal.pgph.0003258)
Supplement: S4 Table — (DOCX) [file pgph.0003258.s006.docx]

**S4 Table. Association between uptake of social/healthcare interventions and no condomless-sex, by age and sex**

|  | **13-19** | | | | **20-35** | | | |
| --- | --- | --- | --- | --- | --- | --- | --- | --- |
|  | **Males** | | **Females** | | **Males** | | **Females** | |
|  | **Unadjusted OR (95% CI)** | **Adjusted OR (95% CI)** | **Unadjusted OR (95% CI)** | **Adjusted OR (95% CI)** | **Unadjusted OR (95% CI)** | **Adjusted OR (95% CI)** | **Unadjusted OR (95% CI)** | **Adjusted OR (95% CI)** |
| **Intervention** |  |  |  |  |  |  |  |  |
| None | 1 | 1 | 1 | 1 | 1 | 1 | 1 | 1 |
| Social only | 2.01 (1.03 -3.90) | 2.04 (1.01 -4.12) | 0.69 (0.09 -5.31) | 0.48 (0.06 -4.14) | 2.32 (0.93 -5.83) | 1.81 (0.70 -4.68) | 3.24 (0.98 -10.68) | 2.33 (0.67 -8.15) |
| Healthcare only | 0.91 (0.37 -2.22) | 1.04 (0.39 -2.76) | 0.05 (0.01 -0.43) | 0.19 (0.02 -1.70) | 0.81 (0.52 -1.27) | 0.84 (0.53 -1.32) | 0.80 (0.44 -1.47) | 0.93 (0.49 -1.76) |
| Multi-level | 1.13 (0.63 -2.03) | 1.28 (0.69 -2.37) | 0.15 (0.02 -1.16) | 0.22 (0.03 -1.85) | 1.19 (0.73 -1.92) | 1.05 (0.63 -1.75) | 1.06 (0.57 -1.98) | 1.20 (0.62 -2.31) |
| **Age group** |  |  |  |  |  |  |  |  |
| 20 - 24 |  |  |  |  | 1 | 1 | 1 | 1 |
| 25 - 29 |  |  |  |  | 0.58 (0.40 -0.85) | 0.58 (0.38 -0.90) | 0.65 (0.49 -0.86) | 0.76 (0.54 -1.06) |
| 30 - 35 |  |  |  |  | 0.53 (0.37 -0.78) | 0.53 (0.34 -0.83) |  |  |
| **Geographic area** |  |  |  |  |  |  |  |  |
| Rural | 1 | 1 | 1 | 1 | 1 | 1 | 1 | 1 |
| Urban | 1.16 (0.82 -1.63) | 1.01 (0.70 -1.46) | 0.78 (0.56 -1.10) | 0.75 (0.50 -1.12) | 1.14 (0.84 -1.56) | 1.25 (0.90 -1.73) | 1.05 (0.78 -1.41) | 1.12 (0.82 -1.54) |
| **Highest educational attainment** |  |  |  |  |  |  |  |  |
| None or Some primary | 1 | 1 | 1 | 1 | 1 | 1 | 1 | 1 |
| Some secondary | 0.12 (0.04 -0.37) | 0.10 (0.03 -0.38) | 0.57 (0.24 -1.33) | 0.64 (0.24 -1.74) | 1.78 (0.75 -4.23) | 1.36 (0.56 -3.33) | 0.53 (0.23 -1.20) | 0.42 (0.18 -0.98) |
| Completed secondary | 0.04 (0.01 -0.16) | 0.04 (0.01 -0.23) | 0.12 (0.05 -0.31) | 0.43 (0.14 -1.33) | 1.37 (0.58 -3.26) | 1.14 (0.47 -2.79) | 0.53 (0.23 -1.19) | 0.49 (0.21 -1.14) |
| **Migration** |  |  |  |  |  |  |  |  |
| Never | 1 | 1 | 1 | 1 | 1 | 1 | 1 | 1 |
| Within PIPSA | 0.41 (0.23 -0.74) | 0.54 (0.29 -1.02) | 0.53 (0.28 -1.00) | 0.71 (0.33 -1.52) | 0.78 (0.47 -1.30) | 0.98 (0.58 -1.66) | 0.85 (0.57 -1.27) | 0.92 (0.60 -1.40) |
| External migration | 0.39 (0.13 -1.16) | 0.50 (0.16 -1.58) | 0.31 (0.15 -0.63) | 0.86 (0.36 -2.07) | 0.88 (0.63 -1.23) | 1.36 (0.92 -2.02) | 0.77 (0.56 -1.06) | 1.04 (0.72 -1.49) |
| **Household wealth index** |  |  |  |  |  |  |  |  |
| Low | 1 | 1 | 1 | 1 | 1 | 1 | 1 | 1 |
| Middle | 0.91 (0.59 -1.41) | 0.81 (0.50 -1.30) | 1.17 (0.77 -1.76) | 1.30 (0.80 -2.11) | 0.82 (0.53 -1.27) | 0.88 (0.57 -1.39) | 1.00 (0.69 -1.46) | 0.92 (0.62 -1.38) |
| High | 1.00 (0.65 -1.54) | 0.92 (0.57 -1.49) | 1.33 (0.87 -2.01) | 1.21 (0.73 -2.00) | 0.82 (0.53 -1.25) | 0.87 (0.56 -1.35) | 0.80 (0.54 -1.17) | 0.76 (0.51 -1.15) |
| Unknown | 0.84 (0.49 -1.46) | 0.76 (0.42 -1.36) | 1.02 (0.60 -1.75) | 0.97 (0.51 -1.85) | 0.84 (0.48 -1.45) | 0.91 (0.52 -1.61) | 0.72 (0.44 -1.17) | 0.75 (0.45 -1.24) |
| **Food insecurity** |  |  |  |  |  |  |  |  |
| No | 1 | 1 | 1 | 1 | 1 | 1 | 1 | 1 |
| Yes | 0.95 (0.63 -1.42) | 1.00 (0.64 -1.54) | 0.48 (0.33 -0.70) | 0.53 (0.33 -0.85) | 0.95 (0.66 -1.37) | 0.88 (0.60 -1.29) | 0.80 (0.57 -1.11) | 0.80 (0.57 -1.14) |
| **Ever had sex, ever been pregnant** |  |  |  |  |  |  |  |  |
| Never | 1 | 1 | 1 | 1 | 1 | 1 | 1 | 1 |
| Ever had sex, never pregnant | 0.06 (0.04 -0.08) | 0.06 (0.04 -0.10) | 0.06 (0.04 -0.10) | 0.08 (0.05 -0.13) | 0.10 (0.05 -0.20) | 0.11 (0.06 -0.23) | 0.13 (0.06 -0.29) | 0.13 (0.06 -0.29) |
| Ever pregnant |  |  | 0.05 (0.03 -0.07) | 0.06 (0.04 -0.10) |  |  | 0.10 (0.05 -0.21) | 0.11 (0.05 -0.25) |
| Unknown | 0.61 (0.07 -4.96) | 0.61 (0.07 -5.10) | 0.05 (0.01 -0.23) | 0.07 (0.01 -0.32) | 0.14 (0.05 -0.41) | 0.17 (0.05 -0.53) | 0.13 (0.03 -0.69) | 0.14 (0.03 -0.77) |
