# Supplementary material for: Uptake of multi-level HIV interventions and HIV-related behaviours among young people in rural South Africa
Source: PLOS Glob Public Health. 2024 May 31;4(5):e0003258. doi: 10.1371/journal.pgph.0003258 (PMC11142690; doi:10.1371/journal.pgph.0003258)
Supplement: S5 Table — (DOCX) [file pgph.0003258.s007.docx]

**S5 Table. Association between uptake of social/healthcare interventions and VMMC, by age**

|  | **Overall** | | **13-19** | | **20-35** | |
| --- | --- | --- | --- | --- | --- | --- |
|  | **Unadjusted OR (95% CI)** | **Adjusted OR (95% CI)** | **Unadjusted OR (95% CI)** | **Adjusted OR (95% CI)** | **Unadjusted OR (95% CI)** | **Adjusted OR (95% CI)** |
| **Intervention** |  |  |  |  |  |  |
| None | 1 | 1 | 1 | 1 | 1 | 1 |
| Social only | 0.76 (0.41 -1.39) | 0.65 (0.32 -1.32) | 0.97 (0.39 -2.44) | 0.98 (0.37 -2.54) | - | - |
| Healthcare only | 0.76 (0.44 -1.33) | 1.08 (0.57 -2.02) | 1.61 (0.39 -6.58) | 2.18 (0.47 -10.18) | 0.68 (0.36 -1.28) | 0.88 (0.43 -1.80) |
| Multi-level | 1.30 (0.77 -2.18) | 1.21 (0.67 -2.19) | 1.75 (0.73 -4.20) | 1.69 (0.68 -4.21) | 0.92 (0.45 -1.88) | 0.86 (0.38 -1.97) |
| **Age group** |  |  |  |  |  |  |
| 13-19 | 1 | 1 |  |  |  |  |
| 20-24 | 0.84 (0.53 -1.35) | 0.61 (0.33 -1.14) |  |  | 1 | 1 |
| 25-29 | 0.92 (0.58 -1.47) | 0.81 (0.40 -1.66) |  |  | 1.09 (0.61 -1.94) | 1.30 (0.66 -2.57) |
| 30-35 | 0.48 (0.28 -0.80) | 0.44 (0.20 -0.96) |  |  | 0.56 (0.30 -1.05) | 0.73 (0.35 -1.54) |
| **Geographic area** |  |  |  |  |  |  |
| Rural | 1 | 1 | 1 | 1 | 1 | 1 |
| Urban | 1.19 (0.85 -1.67) | 1.27 (0.88 -1.82) | 1.42 (0.87 -2.32) | 1.35 (0.82 -2.24) | 1.11 (0.68 -1.81) | 1.19 (0.70 -2.02) |
| **Highest educational attainment** |  |  |  |  |  |  |
| None or Some primary | 1 | 1 | 1 | 1 | 1 | 1 |
| Some secondary | 1.44 (0.71 -2.91) | 1.15 (0.55 -2.39) | 1.09 (0.48 -2.49) | 0.97 (0.41 -2.29) | 2.61 (0.59 -11.52) | 1.82 (0.39 -8.61) |
| Completed secondary | 1.35 (0.63 -2.88) | 1.31 (0.56 -3.06) | 0.44 (0.05 -4.04) | 0.26 (0.03 -2.64) | 3.27 (0.74 -14.38) | 2.40 (0.51 -11.38) |
| **Migration** |  |  |  |  |  |  |
| Never | 1 | 1 | 1 | 1 | 1 | 1 |
| Within PIPSA | 0.88 (0.48 -1.64) | 0.94 (0.47 -1.88) | 0.22 (0.03 -1.73) | 0.16 (0.02 -1.34) | 1.32 (0.64 -2.71) | 1.62 (0.73 -3.59) |
| External migration | 0.59 (0.38 -0.91) | 0.79 (0.43 -1.42) | 0.63 (0.07 -5.45) | 0.89 (0.10 -8.06) | 0.70 (0.41 -1.19) | 0.88 (0.46 -1.68) |
| **Household wealth index** |  |  |  |  |  |  |
| Low | 1 | 1 | 1 | 1 | 1 | 1 |
| Middle | 1.95 (1.21 -3.16) | 2.06 (1.24 -3.41) | 2.15 (1.10 -4.22) | 2.22 (1.11 -4.44) | 1.81 (0.91 -3.61) | 1.69 (0.80 -3.59) |
| High | 1.65 (1.02 -2.65) | 1.63 (0.98 -2.70) | 2.05 (1.06 -3.94) | 2.04 (1.04 -4.02) | 1.33 (0.66 -2.67) | 1.14 (0.53 -2.46) |
| Unknown | 1.05 (0.53 -2.09) | 1.23 (0.60 -2.49) | 1.58 (0.64 -3.93) | 1.54 (0.60 -3.93) | 0.66 (0.22 -1.95) | 0.70 (0.22 -2.21) |
| **Food insecurity** |  |  |  |  |  |  |
| No | 1 | 1 | 1 | 1 | 1 | 1 |
| Yes | 0.92 (0.62 -1.37) | 0.99 (0.64 -1.51) | 1.11 (0.62 -1.99) | 1.10 (0.59 -2.03) | 0.84 (0.48 -1.46) | 0.90 (0.49 -1.65) |
| **Ever had sex** |  |  |  |  |  |  |
| No | 1 | 1 | 1 | 1 | 1 | 1 |
| Yes | 0.83 (0.59 -1.17) | 1.13 (0.68 -1.88) | 1.62 (0.93 -2.81) | 1.65 (0.91 -2.98) | 0.60 (0.26 -1.40) | 0.57 (0.23 -1.39) |
