# Supplementary material for: Uptake of multi-level HIV interventions and HIV-related behaviours among young people in rural South Africa
Source: PLOS Glob Public Health. 2024 May 31;4(5):e0003258. doi: 10.1371/journal.pgph.0003258 (PMC11142690; doi:10.1371/journal.pgph.0003258)
Supplement: S6 Table — (DOCX) [file pgph.0003258.s008.docx]

**S6 Table. Association between uptake of interventions and HIV-related behaviours among adolescents and young adults**

|  | **No condomless sex** | | | **VMMC** | | |
| --- | --- | --- | --- | --- | --- | --- |
| **Intervention** | **n (%)** | **OR (95% CI)** | **aOR (95% CI)** | **n (%)** | **OR (95% CI)** | **aOR (95% CI)** |
| **Safe spaces** |  |  |  |  |  |  |
| No | 690 (69.1) | 1 | 1 |  |  |  |
| Yes | 422 (78.7) | 1.66 (1.30 - 2.12) | 1.05 (0.80 - 1.38) |  |  |  |
| **Mentor program** |  |  |  |  |  |  |
| No | 834 (70.6) | 1 | 1 |  |  |  |
| Yes | 277 (76.7) | 1.54 (1.16 - 2.05) | 0.97 (0.71 - 1.32) |  |  |  |
| **Social assets** |  |  |  |  |  |  |
| No | 935 (71.8) | 1 | 1 |  |  |  |
| Yes | 176 (75.9) | 1.23 (0.89 - 1.71) | 0.78 (0.55 - 1.10) |  |  |  |
| **Business skills training** |  |  |  |  |  |  |
| No | 2083 (61.6) | 1 | 1 | 164 (20.4) | 1 | 1 |
| Yes | 246 (71.9) | 1.60 (1.25 - 2.04) | 0.93 (0.70 - 1.24) | 7 (28.0) | 1.52 (0.63 - 3.71) | 1.45 (0.58 - 3.66) |
| **Financial literacy** |  |  |  |  |  |  |
| No | 2004 (60.8) | 1 | 1 | 151 (20.2) | 1 | 1 |
| Yes | 327 (75.7) | 2.01 (1.59 - 2.53) | 1.29 (1.00 - 1.68) | 20 (24.7) | 1.30 (0.76 - 2.22) | 1.11 (0.62 - 2.00) |
| **Parenting program** |  |  |  |  |  |  |
| No | 1284 (81.9) | 1 | 1 | 69 (22.9) | 1 | 1 |
| Yes | 500 (83.5) | 1.11 (0.87 - 1.43) | 1.10 (0.85 - 1.43) | 23 (25.0) | 1.12 (0.65 - 1.94) | 1.16 (0.66 - 2.04) |
| **Violence prevention** |  |  |  |  |  |  |
| No | 2071 (61.2) | 1 | 1 | 163 (20.5) | 1 | 1 |
| Yes | 261 (75.4) | 1.94 (1.51 - 2.61) | 1.06 (0.79 - 1.41) | 8 (23.5) | 1.20 (0.53 - 2.69) | 1.06 (0.46 - 2.46) |
| **Post-violence care** |  |  |  |  |  |  |
| No | 2235 (63.4) | 1 | 1 | 165 (20.9) | 1 | 1 |
| Yes | 95 (47.5) | 0.52 (0.39 - 0.70) | 1.03 (0.74 - 1.43) | 6 (15.0) | 0.67 (0.28 - 1.62) | 0.73 (0.29 - 1.83) |
| **STI screening & treatment** |  |  |  |  |  |  |
| No | 2228 (63.9) | 1 | 1 | 156 (20.2) | 1 | 1 |
| Yes | 103 (42.9) | 0.42 (0.33 - 0.55) | 0.74 (0.55- 1.01) | 15 (25.0) | 1.31 (0.71 - 2.42) | 1.88 (0.97 - 3.64) |
| **VMMC** |  |  |  |  |  |  |
| No | 729 (55.1) | 1 | 1 |  |  |  |
| Yes | 354 (72.1) | 2.10 (1.67 - 2.63) | 1.31 (1.00 - 1.72) |  |  |  |
| **Adolescent & youth friendly services** |  |  |  |  |  |  |
| No | 1884 (72.6) | 1 | 1 | 108 (22.0) | 1 | 1 |
| Yes | 206 (67.8) | 0.79 (0.62 - 1.02) | 0.77 (0.58 - 1.03) | 13 (28.3) | 1.39 (0.71 - 2.74) | 1.36 (0.66 - 2.79) |
