# Supplementary material for: Uptake of multi-level HIV interventions and HIV-related behaviours among young people in rural South Africa
Source: PLOS Glob Public Health. 2024 May 31;4(5):e0003258. doi: 10.1371/journal.pgph.0003258 (PMC11142690; doi:10.1371/journal.pgph.0003258)
Supplement: S7 Table — (DOCX) [file pgph.0003258.s009.docx]

**S7 Table. Factors associated with lost to follow-up among adolescents and young adults enrolled in the cohort studies.**

| **Factor** | **Unadjusted OR (95% CI)** | **Fully Adjusted OR (95% CI)** |
| --- | --- | --- |
| **Sex** |  |  |
| Male | 1 | 1 |
| Female | 0.78 (0.68 -0.90) | 0.90 (0.74 -1.11) |
| **Age group** |  |  |
| 13-19 | 1 | 1 |
| 20-24 | 2.19 (1.84 -2.61) | 1.28 (1.02 -1.62) |
| 25-29 | 2.01 (1.66 -2.44) | 1.00 (0.75 -1.35) |
| 30-35 | 2.76 (2.16 -3.52) | 1.17 (0.83 -1.65) |
| **Urbanicity** |  |  |
| Rural | 1 | 1 |
| Peri-urban | 1.13 (0.97 -1.30) | 1.03 (0.87 -1.20) |
| **Household wealth index** |  |  |
| Low | 1 | 1 |
| Middle | 0.74 (0.61 -0.90) | 0.72 (0.58 -0.90) |
| High | 1.05 (0.87 -1.26) | 1.00 (0.82 -1.23) |
| Unknown | 1.25 (0.99 -1.57) | 1.23 (0.96 -1.57) |
| **Migration** |  |  |
| Never | 1 | 1 |
| Within PIPSA | 1.19 (0.93 -1.53) | 0.87 (0.66 -1.15) |
| External migration | 2.03 (1.73 -2.39) | 1.26 (1.01 -1.57) |
| **Highest educational attainment** |  |  |
| None or some primary | 1 | 1 |
| Some secondary | 1.28 (0.90 -1.83) | 1.21 (0.83 -1.75) |
| Completed secondary | 2.82 (1.96 -4.07) | 1.87 (1.25 -2.79) |
| **Food insecurity** |  |  |
| No | 1 | 1 |
| Yes | 0.91 (0.76 -1.08) | 0.83 (0.68 -1.00) |
| **Ever had sex, ever pregnant** |  |  |
| Never had sex | 1 | 1 |
| Ever sex, never pregnant | 2.63 (2.23 -3.11) | 1.85 (1.45 -2.38) |
| Ever pregnant | 1.84 (1.52 -2.24) | 1.41 (1.04 -1.91) |
| Unknown | 1.88 (1.15 -3.06) | 1.00 (1.00 -1.00) |
| **No condomless sex in past 12mo** |  |  |
| No | 1 | 1 |
| Yes | 0.60 (0.52 -0.70) | 1.05 (0.87 -1.26) |
